# Oral and Facial Surgery

**Published:** 1893-08

**Authors:** 


### ORAL AND FACIAL SURGERY.

To the general practitioner of medicine, this specialty is almost unknown, and by the surgeon, unrecognized, as he acknowledges no specialty in what really is a stupendous field. Some important specialties in surgery, however, have long since been recognized, and even almost reverenced, for the great advancement they have made in their special lines, and it is to these men, who had the courage to carry out their convictions, that the world owes unlimited obligations for the great advancement made in the practice of medicine and surgery, and it is my firm belief to-day, that just such a career awaits the Oral Surgeon; although, perhaps, unacknowledged, his need must soon be felt. Surgery, like medicine, is too broad and too complicated to be compassed in its every differentiation, to the degree required in these days of perfected technique, by any one mind. Every department requires for the attainment of the best results concentration of the entire thought, upon the particular set of organs involved, almost to the exclusion of the remainder of the body, bearing in mind, only the fact, that other parts may exert an influence by contiguity, or by physical, or physiological relations, or may become involved by reflex or circulatory action. This concentration and study of minutiæ with the daring experimental work, begotten of more thorough knowledge thus gained, are the secret of the present status of the various specialties in medicine, and of the vastly better results of practice by specialists now than in former times, when each practitioner endeavored to cover the entire field.

It is the little things, which, because of the littleness, are unrecognized and their proper treatment overlooked, that often lead to the most confusing complications. In view of all this, does it not look reasonable that a man, devoting his entire time and energy to the study and practice of one particular branch, becomes more proficient than he who attempts to grasp the whole subject? Prejudice must be put aside, and the advancement of science allowed free scope.

It is not with the view of condemning the general practitioner that I shall refer to some cases, but to bring forth the necessity for a fuller knowledge on the subject and lead him to a closer observation of the mouth and its diseases, by which he will be more speedily enabled to reach a diagnosis, and thus incur a cure of his cases, which often for months baffle his greatest skill. No one kind of treatment will cure any one, or all kinds of diseases. This is exemplified in the following case, where a patient had for six years been under constant treatment for facial neuralgia, and had gone the rounds of specialists from the dentist to the physician, from drugs to electricity, and all without permanent relief, till at last the Oral specialist was thought of. He, on examination, found a hypoplasia condition existing in a tooth which, upon opening into, revealed a calcification of the pulp, the removal of which instantly and completely cured the malady. Many cases of this wearisome affection are relieved, and sometimes cured by the use of internal treatment, but when the cause can be singled out and removed, as is the case in about 90 per cent. of all cases of neuralgia within the regions of distribution of the Trigeminous nerve and those subject to reflex irritation, it should be done. Is it not the need of knowledge of the obscure and remote diseases of the teeth and jaws which leads physicians and surgeons to rash measures, or to suspect other, and innocent organs, in being responsible for all their anxiety and the patient's suffering?

The fact that the mouth is the index of the physical state of the system, should not be lost sight of. Our most sensitive sense is the sense of smell; few observe their own offensiveness, and as few are privileged to tell them of it. The part of the body, where most germs of disease are found, is the human mouth. This may account for most, if not all, of the maladies to which the body is heir. Have you stopped to think why some people have an offensive breath, and others have not, and why it is present to-day, and not tomorrow? It is not always traceable to an overloaded stomach and indigestion, but more often to the destructive ravages of some of the seventy different kinds of germs which infest the oral cavity. While the treatment of these diseases has been left almost entirely to the care of the dentist, few there are among them whose knowledge extends to anything beyond what directly applies to the restoration of the teeth; therefore the opinion of the family dentist is not always to be relied upon, whether the cause of reflected trouble, such as neuralgia, is traceable to the teeth, or jaws, and therefore I predict that the time is close at hand when as thorough a knowledge of the mouth and all its details will be required of physicians, as is the knowledge of anatomy and physiology at the present time.

We do not condemn the physician for the lack of knowledge, for his opportunities for obtaining it have been limited, but what we do want is for him to take advantage of such as there are,—to think and refer such cases to the various specialists, to relieve his confiding and dependent patient. Perhaps the greatest compliment a physician can pay himself is to acknowledge his inability to cure such cases, and to take them to others for special study and treatment.

We are only in the center of scientific research and advancement, and he who thinks this the flood-tide of knowledge is groping in the dark. Within the past two score of years, surgery has advanced like a great, victorious army, and has carried its standard farther to the front than, perhaps, any other department of science, and also I hope, buried the many, petty jealousies which were once responsible for so much neglect, by educating us to be broader and better men.—*The Post Graduate.*

---

